# Supplementary material for: A social system to disperse the irrigation start date based on the spatial public goods game
Source: PLoS One. 2023 May 24;18(5):e0286127. doi: 10.1371/journal.pone.0286127 (PMC10208473; doi:10.1371/journal.pone.0286127)
Supplement: S1 Appendix — (DOCX) [file pone.0286127.s001.docx]

**Appendix**

We performed simulations under the condition that the cooperation cost $C_{co}$, is 10000–100000 [yen/ha/year] when the average degree of the farmer's information reference network is 8, 12, and 20. As a result, despite changes in the cooperation cost, the results for the proportion of cooperators and the standard deviation of irrigation starting dates remained unchanged (Figs. A1–A6). Therefore, we conclude that the results are robust to changes in the cooperation cost under these conditions of average degree, as well as the condition in which the average degree is four (Figs. 8 and 9).


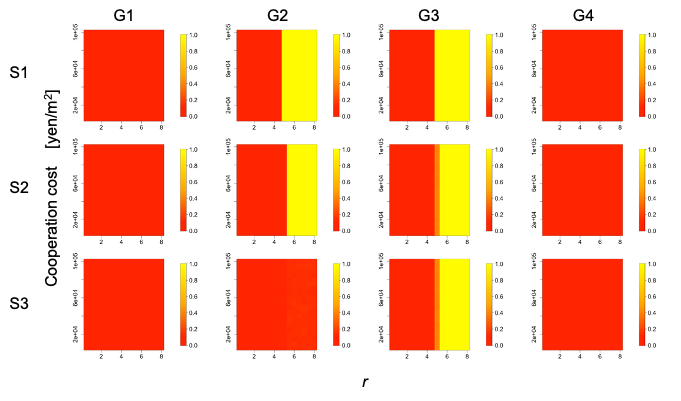


**Fig. A1. Relationships among *r*, cooperation costs, and the proportion of cooperators (the average degree is eight)**

Columns (G1) – (G4) of the panel show the results for group conditions 1–4. Rows (S1) – (S3) of the panel display the condition of complete information without exogenous preference (scenario 1), the condition of incomplete information without exogenous preference (scenario 2), and the condition of incomplete information with exogenous preference (scenario 3), respectively. Color indicates the proportion of cooperators. This result is for year 200 and $C_{co}$= 10000 in the simulation.


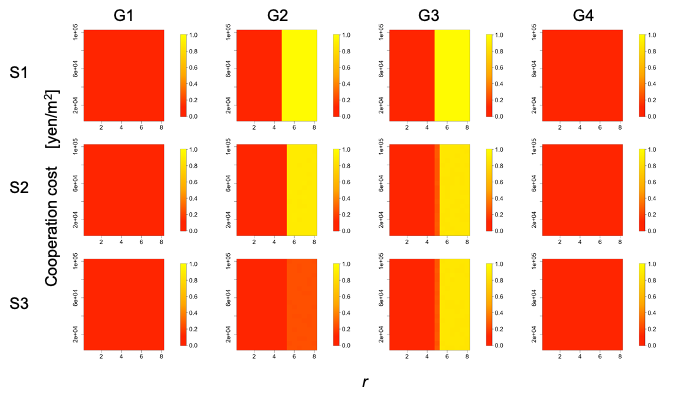


**Fig. A2. Relationships among *r*, cooperation costs, and the proportion of cooperators (the average degree is twelve)**

The reading of the figure is the same as that in Figure A1.


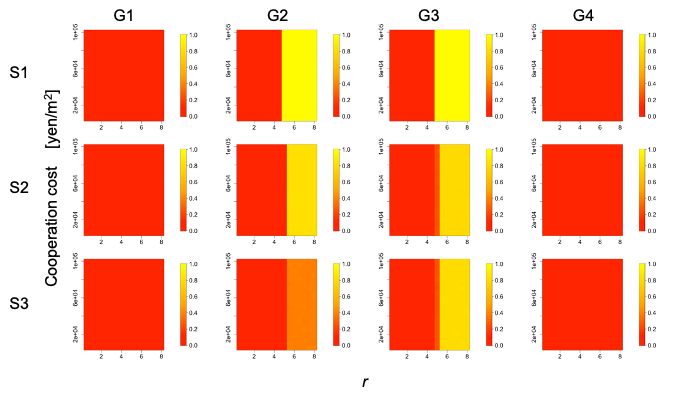


**Fig. A3. Relationships among *r*, cooperation costs, and the proportion of cooperators (the average degree is twenty)**

The reading of the figure is the same as that in Figure A1.


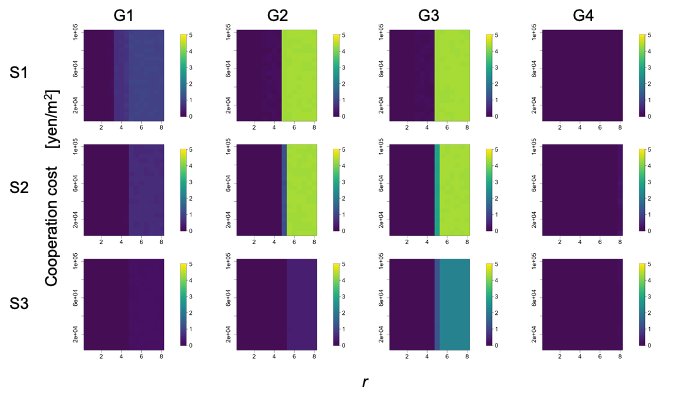


**Fig. A4. Relationships among *r*, cooperation costs, and standard deviation of irrigation starting dates (the average degree is eight).**

Columns (G1) – (G4) of the panel show the results for group conditions 1–4. Rows (S1) – (S3) of the panel display the condition of complete information without exogenous preference (scenario 1), the condition of incomplete information without exogenous preference (scenario 2), and the condition of incomplete information with exogenous preference (scenario 3), respectively. The color indicates the standard deviation of the irrigation start dates. This result is for year 200 and $C_{co}$= 10000 in the simulation.


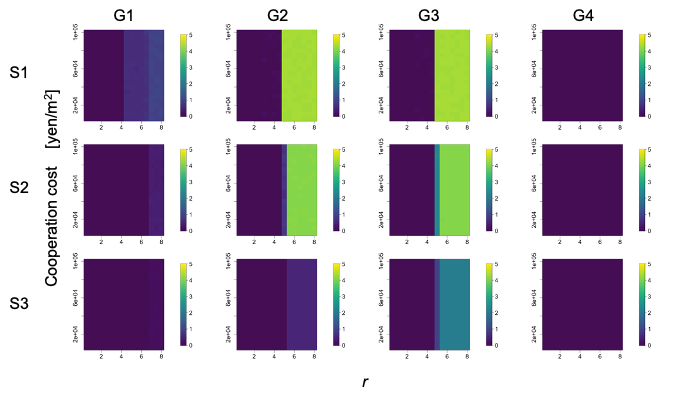


**Fig. A5. Relationships among *r*, cooperation costs, and standard deviation of irrigation starting dates (the average degree is twelve).**

The reading of the figure is the same as that in Figure A4.


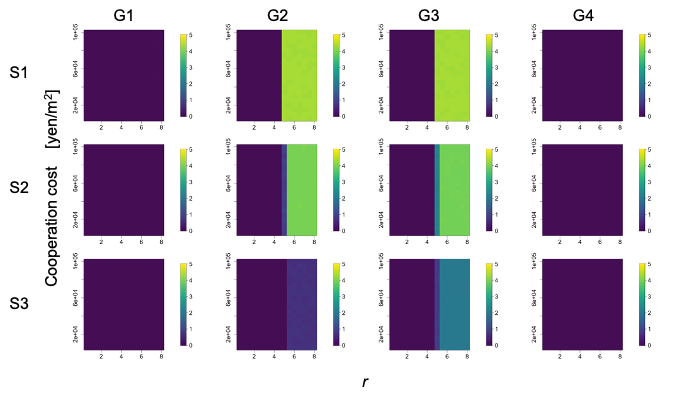


**Fig. A6. Relationships among *r*, cooperation costs, and standard deviation of irrigation starting dates (the average degree is twenty).**

The reading of the figure is the same as that in Figure A4.
